# Feature ranking and rank aggregation for automatic sleep stage classification: a comparative study

**DOI:** 10.1186/s12938-017-0358-3

**Published:** 2017-08-18

**Authors:** Shirin Najdi, Ali Abdollahi Gharbali, José Manuel Fonseca

**Affiliations:** 1 Computational Intelligence Group of CTS/UNINOVA, Caparica, Portugal; 2 Faculdade de Ciências e Tecnologia, Universidade Nova de Lisboa, Campus da Caparica, Quinta da Torre, 2829-516 Caparica, Portugal

**Keywords:** Sleep stage classification, Feature selection, Rank aggregation, Feature ranking, Polysomnography, Biomedical signal processing, *k*-NN, Neural network

## Abstract

**Background:**

Nowadays, sleep quality is one of the most important measures of healthy life, especially considering the huge number of sleep-related disorders. Identifying sleep stages using polysomnographic (PSG) signals is the traditional way of assessing sleep quality. However, the manual process of sleep stage classification is time-consuming, subjective and costly. Therefore, in order to improve the accuracy and efficiency of the sleep stage classification, researchers have been trying to develop automatic classification algorithms. Automatic sleep stage classification mainly consists of three steps: pre-processing, feature extraction and classification. Since classification accuracy is deeply affected by the extracted features, a poor feature vector will adversely affect the classifier and eventually lead to low classification accuracy. Therefore, special attention should be given to the feature extraction and selection process.

**Methods:**

In this paper the performance of seven feature selection methods, as well as two feature rank aggregation methods, were compared. Pz-Oz EEG, horizontal EOG and submental chin EMG recordings of 22 healthy males and females were used. A comprehensive feature set including 49 features was extracted from these recordings. The extracted features are among the most common and effective features used in sleep stage classification from temporal, spectral, entropy-based and nonlinear categories. The feature selection methods were evaluated and compared using three criteria: classification accuracy, stability, and similarity.

**Results:**

Simulation results show that MRMR-MID achieves the highest classification performance while Fisher method provides the most stable ranking. In our simulations, the performance of the aggregation methods was in the average level, although they are known to generate more stable results and better accuracy.

**Conclusions:**

The Borda and RRA rank aggregation methods could not outperform significantly the conventional feature ranking methods. Among conventional methods, some of them slightly performed better than others, although the choice of a suitable technique is dependent on the computational complexity and accuracy requirements of the user.

## Background

Sleep occupies a significant part of human life. Therefore, the accurate diagnose of sleep-related disorders is of great importance in sleep research. Sleep is a particular condition of the nervous system with noticeable features and brain activity phases. Although most people think that sleep is a passive and constant process, as a matter of fact, sleep is an active state. Human bodies move frequently during the night and the human brain is sometimes even more active than in the waking state [1]. Normal human sleep generally consists of two distinct stages with independent functions known as non-rapid eye movement (NREM) and rapid eye movement (REM) sleep. In their ideal situation, NREM and REM states alternate regularly, each cycle lasting 90 min on average. According to the American Academy of Sleep Medicine (AASM) [2], NREM is subdivided into three stages: stage 1 or light sleep, stage 2 and stage 3 or slow wave sleep (SWS). The evolution of sleep stages is complemented by gradual changes in many behavioral and physiological occurrences. Sleep stages are commonly classified using multiple simultaneous physiologic parameters during sleep named Polysomnography (PSG) in a clinic or hospital environment. A collection of rules has been identified in the AASM to guide the practitioners. However, the visual process of sleep stage classification is time-consuming, subjective and costly. In order to improve the accuracy and efficiency of the sleep stage classification, researchers have been trying to develop automatic classification algorithms. The automatic sleep stage classification mainly consists of three steps: pre-processing, feature extraction and classification [3]. In the feature extraction stage, several temporal, spectral and nonlinear features are extracted from PSG signals. Nevertheless, some of these features may be irrelevant or have high mutual correlation increasing the complexity of the model without any real benefit. To face this challenge, feature selection and dimensionality reduction methods have been utilized.

In principle, a feature selection method has been used with the aim of selecting a subset of features in a way that the classifier can distinguish the differences between various classes of input data more effectively. The advantages of using feature selection methods make it an essential requirement for many classification applications. Reaching a more compact and simple model is the most important advantage offered by the feature selection process, that can reduce the necessary computational time for the classifier. Also, enhancing the generalization ability, increasing the classification power through reduced overfitting level, less storage memory and simplified visualization are further benefits of feature selection in classification tasks. Several different types of feature selection methods exist in the literature. Among them, the most common methods are divided into three main categories: filter, wrapper and embedded methods. Filter methods perform feature selection by considering some intrinsic characteristics of the data and usually provide a rank or a score for each feature. Low scored features will be removed experimentally or according to a predefined threshold. Filter methods, besides being simple and fast, are independent of the classifier.

Wrapper methods on the other hand, embed a search algorithm in the space of possible features subsets. Then, various subsets are produced and evaluated by training and testing with the specific classification algorithm. Since, the number of possible subsets grows exponentially with the number of features, heuristic search algorithms are used for finding the optimal feature subset. The high computational complexity and the risk of over fitting are its main disadvantages. The main benefits of wrapper methods over filter methods are taking into account feature dependencies and interaction between the selected subset and the specific classification method.

Embedded methods integrate the optimal feature subset selection with the classification algorithm. They have less computational complexity compared to wrapper methods. The results of both wrapper methods and embedded methods are classifier-specific.

In sleep stage classification, filter methods are more common than wrapper and embedded methods. Among the filter methods fast correlation based filter (FCBF), Fisher score, ReliefF, Chi square (Chi2), information gain (IG), conditional mutual information maximization (CMIM), minimum redundancy maximum relevance (MRMR) algorithms and R-square [4–7] are the most preferred ones. In addition to the traditional methods, a new filter method called ‘Mahal’ is proposed in [8] for facing the challenge of feature selection in small datasets with a large number of features for sleep stage classification. On the other hand, sequential feature selection algorithms including sequential forward selection (SFS) and sequential backward selection (SBS) are the most common wrapper methods used in the automatic sleep stage classification [9, 10]. Statistical hypothesis testing methods are also used in sleep stage classification applications for feature selection and dimensionality reduction. Examples of these methods are *t* test, ANOVA and Kruksal–Wallis test which are used for three different purposes: dimensionality reduction, feature selection and assessment of the discriminative capability of the selected feature set. To the best of our knowledge, there are no studies for comparing the performance of various feature selection methods from the same category in sleep stage classification. The studies done so far usually choose feature selection methods from different categories. For example in [11], one filter and three wrapper methods are used and the results are compared. Therefore, there is a need for comprehensive comparison of feature selection methods from the same category.

As mentioned before, feature ranking techniques provide a ranked list of features. Different feature ranking techniques may produce different rankings according to their specific criteria for assessing features and there is no universal ranking algorithm that considers all the measures. Therefore, motivated by ensemble methods in supervised learning [12], rank aggregation methods are proposed to combine different feature ranking methods and achieve more stable ranked feature lists with similar or even higher classification performance [13, 14]. In order to perform ensemble feature selection, one needs to decide on the method to aggregate the results from different ranking methods. There are many rank aggregation approaches from the very simple ones to some more complex [14]. To the best of our knowledge, there are no studies done on feature selection based on rank aggregation methods in the sleep stage classification area.

In this paper different feature ranking and rank aggregation methods were compared within the sleep stage classification context. The main contributions of this paper are listed below:

1. A comprehensive feature set including Itakura Spectral Distance (ISD) [15] was extracted from PSG signals,
2. Similarity and stability of different feature ranking and rank aggregation methods were assessed,
3. Classification performance of different feature ranking and rank aggregation methods was compared.

In this work, we present the extension of the results published in [4]. The paper is organized as follows: In the next section (“Methods”) the database, pre-processing, extracted features, feature selection techniques and classification algorithms will be described. In the following section related results will be shown. Discussion of the obtained results will be presented in the next section. On the last section, the conclusions and future work directions are presented.

## Methods

**Fig. 1:**
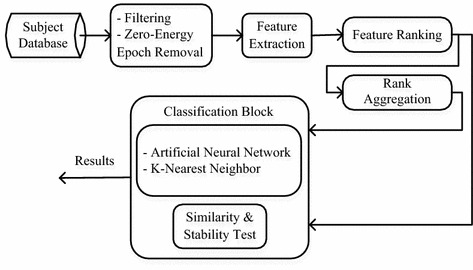
Block diagram for comparison of feature selection methods

In this section the sleep stage classification methodology used in this work is described in detail. Figure 1 shows the block diagram of the proposed algorithm for comparing the feature selection methods.

### Data

The data used in this study was obtained from Physionet Sleep-EDF Expanded Database [15]. The collection of data in this database comes from two studies. PSG recordings of the first study are named SC files (SC = Sleep Cassette). PSG recordings of the second study are named ST files (ST = Sleep Telemetry). In our simulations, we didn’t use SC files, since EMG data for first study was a zero-amplitude or no-data recording. Therefore, we used ST files which are a collection of 22 PSG signals recorded in the hospital during two nights for about 9 h. Except for slight difficulty in falling asleep, subjects were healthy without any sleep related medication. The data were segmented into 30-s epochs and all epochs were scored according to R&K guidelines for human sleep staging. These recordings include EEG (Fpz-Cz and Pz-Oz), EOG (horizontal), submental chin EMG, together with the corresponding hypnograms.

Through careful analysis ST recordings, a number of issues were detected that made some of recordings unsuitable for being used in the evaluations. These issues are as follows:

- Lack of stage 4 (according to R&K guidelines),
- Artifacts such as severe movement or sensor misconnection,
- Unsynchronized EEG data and hypnogram,
- Lack of stage 3 epochs,
- Severely corrupted EEG data.

As a results, six recordings were selected out of twenty-two and the corresponding hypnograms were converted from R&K to AASM. Pz-Oz channel EEG together with submental chin EMG and horizontal EOG each sampled at 100 Hz were used in the evaluations. Table 1 illustrates the number of stages available per subject.

**Table 1:** Summary of the data provided by six selected subjects

	Wake	REM	S1	S2	SWS
Subject #1	146	122	101	527	136
Subject #2	41	159	71	351	284
Subject #3	85	226	120	392	180
Subject #4	40	143	47	266	152
Subject #5	149	80	102	428	218
Subject #6	131	142	135	378	198

### Pre-processing

Artifact free data is necessary for guaranteeing the reliability of sleep stage classification algorithms. In this work, the epochs with zero-energy were automatically detected and removed. The zero-energy epochs can appear due to the possible failure of recording device. Then, the EEG and EOG signals were band-pass filtered in the frequency interval of 0.3–35 Hz. This interval was selected according to the recommendations of AASM. For filtering, wavelet multi-level decomposition and reconstruction was used. This filtering technique has a high fidelity to the original wide-band signal in contrast to the Butterworth filtering that produces a highly distorted “valley” shape [16].

### Feature extraction and normalization

In order to explore the information contained in PSG recordings, a set of features were extracted from EEG, H_EOG and submental chin EMG of each subject. This feature set includes 49 features that can be categorized into time, frequency, joint time–frequency domain, entropy-based and nonlinear types. As summarized in Table 2. In the following, information will be provided about the different features used in this study and their brief description.

**Table 2:** Summary of the features extracted from PSG recordings

Signal	Category	Feature name
EEG	Time domain (F1 to F12)	Statistical features (minimum value, maximum value, arithmetic mean, standard deviation, variance, skewness, kurtosis, median), zero-crossing rate, Hjorth parameters (activity, mobility and complexity) [32]
	Time–frequency domain (F13 to F26)	Features extracted from wavelet packet coefficients including energy of *α*, *δ*, *β1*, *β2*, *θ* and spindle bands, total energy of all bands, energy ratio of ($\frac{\alpha }{\delta + \theta }$, $\frac{\delta }{\alpha + \theta }$, $\frac{\theta }{\alpha + \delta }$, $\frac{\delta }{\theta }$, $\frac{\alpha }{\theta }$), statistical features (mean and standard deviation of coefficients in all of the bands)
	Entropy (F27 to F30)	Spectral entropy, Rényi entropy, approximate entropy, permutation entropy [32]
	Non-linear (F31 to F36)	Petrosian fractal dimension, teager energy, energy, mean curve length, hurst exponent [32], ISD
EOG	Time domain (F37 to F41)	Mean, maximum, standard deviation, skewness, kurtosis [58]
	Non-linear (F42)	Energy [58]
EMG	Frequency domain (F43 to F46)	Total power in the EMG frequency spectrum, statistical features of EMG frequency spectrum (maximum, mean, standard deviation) [58]
	Non-linear (F47 to F49)	Energy, ratio of the EMG Signal energy for the current epoch and previous epoch, ratio of the EMG signal energy for the current epoch and next epoch [58]

- Statistical features (F1 to F8, F37 to F41, and F44 to F46): Understanding the evolution of PSG signals as stochastic processes can provide valuable information regarding the sleep stage classification. In this study, the simple and most common statistical features [17] including mean, median, maximum and minimum values, skewness and kurtosis of each EEG, EOG and EMG epoch are used according to Table 2.
- Zero crossing rate (F9): Zero crossing rate (ZCR) is simple and at the same time very effective feature especially in sleep stage classification. ZCR counts the signals sign-change points on a segment of a signal. In this paper, the length of this segment is 30 s.
- Hjorth parameters (F10 to F12): In 1960, Bo Hjorth [18] proposed normalized slope detectors (NSD) as indicators of statistical properties of a signal in time domain. NSDs include three features: activity, mobility and complexity. These features are widely used in the analysis and characterization of EEG. Hjorth’s NSD are calculated as shown in Table 3 with *σ*_0_ representing the variance of the signal, *σ*_1_ the variance of the first derivate and *σ*_2_ the variance of the second derivate of the signal.

**Table 3:** Hjorth parameters

Feature name	Formula
Hjorth activity	*σ*_0_^2^
Hjorth mobility	*σ*_1_/*σ*_1_
Hjorth Complexity	$\sqrt {({{\sigma_{2} } \mathord{\left/ {\vphantom {{\sigma_{2} } {\sigma_{1} )^{2} }}} \right. \kern-0pt} {\sigma_{1} )^{2} }}} - \left( {{{\sigma_{1} } \mathord{\left/ {\vphantom {{\sigma_{1} } {\sigma_{0} }}} \right. \kern-0pt} {\sigma_{0} }}} \right)^{2}$
- Wavelet based features (F13 to F26): In order to analyze the stochastic nature of EEG, we chose the wavelet packet (WP) analysis since it provides a valuable joint time–frequency domain analysis. In clinical applications, four main brain rhythms are associated with different states of sleep, including Delta (0–3.99 Hz), Theta (4–7.99 Hz), Alpha (8–13 Hz) and Beta (>13 Hz) [2]. According to the scheme proposed in [19], a WP tree with 7 decomposition levels was suitable to estimate the necessary frequency bands of EEG rhythms with adequate accuracy. Then, features F13 to F26 were extracted from the corresponding WP coefficients according to descriptions in Table 2.
- Spectral entropy (F27): Spectral entropy, as a technique for measuring the irregularity of EEG, is calculated by the entropy of the power spectrum. Suppose *P* is the normalized power spectrum of EEG in a predefined frequency range, [*f*_1_, *f*_2_] and $\sum {P_{i} = 1}$, the spectral entropy is calculated as:
  1 $$ H_{sp} = - \sum\limits_{{f_{1} }}^{{f_{2} }} {P_{i} \log P_{i} } $$
  In this study, *H*_*sp*_ is used in the following normalized form:
  2 $$ SpEn = \frac{{H_{sp} }}{{\log N_{f} }} $$
  where *N*_*f*_ is the number of frequency bins in the frequency range [*f*_1_, *f*_2_] [20, 21].
- Rényi entropy (F28): In 1960, Alfréd Rényi introduced Rényi’s general notion of entropy [22]. Since, Rényi Entropy unites several distinct entropy measures, it turned out to be theoretically interesting and found many applications in various research areas such as pattern recognition [23] and biomedicine [24]. Suppose *P*_*x*_(*X*) is probability distribution of random variable *X*. The Rényi entropy of order *α* for *X* is defined as:
  3 $$ H_{\alpha } (X) = \frac{1}{1 - \alpha }\log \sum\limits_{x} {P_{x} (X)^{\alpha } } $$
- Approximate entropy (F29): Approximate entropy is regarded as a measure of the randomness or equivalently regularity of a time series. Considering that time series with repetitive patterns are more predictable than those without repetitive patterns, approximate entropy reflects the likelihood that similar patterns existing in a time series will not be followed by more patterns from the same type [25, 26].
  For calculating approximate entropy two parameters need to be predefined: first the pattern length *m* and second the similarity threshold *r*. Given the time series $\,\left\{ {x_{i} } \right\}_{i = 1 \ldots N}$, a sequence of vectors ${\mathbf{x}}\left( 1 \right)$ through ${\mathbf{x}}(N - m + 1)$ is formed in which $\,{\mathbf{x}}(i) = [x(t), \ldots ,x(t + m - 1)]$. Two vectors ${\mathbf{x}}(i)$ and ${\mathbf{x}}(j)$ are similar if their distance is less than *r*. The distance between two patterns is defined as the maximum difference between their corresponding components. Then, $C_{i}^{m} (r)$ is defined as:
  4 $$ \begin{aligned} C_{i}^{m} (r) = \frac{{{\text{No}} . {\text{ of }}j \le N - m + 1}}{N - m + 1} \hfill \\ \quad \quad \quad \,{\text{for }}d[x(i),x(j)] \le r \hfill \\ \end{aligned} $$
  where $C_{i}^{m} (r)$ expresses the patterns regularity of length *m* with a threshold value of *r*. Finally, approximate entropy is defined as [27]:
  5 $$ \begin{aligned} ApEn(m,r,N) = \frac{1}{N - m + 1}\sum\limits_{i = 1}^{N - m + 1} {\ln (C_{i}^{m} )} \hfill \\ \quad \quad \quad \quad \quad \quad \quad - \frac{1}{N - m}\sum\limits_{i = 1}^{N - m} {\ln (C_{i}^{m} )} \hfill \\ \end{aligned} $$
- Permutation entropy (F30): Permutation entropy was proposed by Bandt et al. [28] and is a simple complexity measure, that can be applied to any type of time series including regular, chaotic, noisy and time series from reality. In mathematical terms, consider that a time series is $\left\{ {x_{t} } \right\}_{t = 1 \ldots T}$. Through an embedding procedure, a set of vectors ${\mathbf{X}}_{t} = [x_{t} ,x_{t + 1} , \ldots ,x_{t + m} ]$ with the embedding dimension *m* is formed. Then, **X**_*t*_ is arranged in an increasing order. There will be $m!$ different order patterns*π*, also called permutations. If $f(\pi )$ denotes the frequency of permutation in the time series, then its relative frequency would be:
  6 $$ p(\pi ) = \frac{f(\pi )}{T - (m - 1)} $$
  Therefore, the permutation entropy is defined as:
  7 $$ H_{p} (m) = - \sum {p(\pi )log} p(\pi ) $$
  where the sum runs over all $m!$ permutations [28, 29].
- Petrosian fractal dimension (F31): The fractal dimension has been widely used in the characterization of nonstationary biomedical signals like EEG for several applications in order to measure the complexity of sleep EEG. Petrosian algorithm can be used for a fast computation of fractal dimension by means of transforming the signal into a binary sequence [30]. Petrosian fractal dimension is calculated using the following formula:
  8 $$ FD_{Petrosian} = \frac{{\log_{10} N}}{{\log_{10} N + \log_{10} (\frac{N}{{N + 0.4N_{\Delta } }})}} $$
  In which *N* is the length of the EEG signal and *N*_Δ_ is the number of sign changes in the derivative of the signal.
- Teager energy (F32): Teager energy operator has been proved to be very useful in analysing signals from the energy point of view. It is defined as:
  9 $$ \Psi (x(t)) = \dot{x}^{2} (t) - x(t)\ddot{x}(t) $$
  in continuous form, where $\dot{x}(t)$ is the first derivative and $\ddot{x}(t)$ is the second derivative of *x*. The discrete form of Teager energy is [31]:
  10 $$ \Psi \left( {x[n]} \right) = x^{2} [n] - x[n - 1]x[n + 1] $$
- Energy (F33, F42, and F47 to F49): Energy is calculated as the average sum of the squares of all samples in a signal segment. Energy value of a signal increases with the increase of activity in the signal [32]. According to Table 2, both energy and energy ratio of different epochs of PSG recordings were used in this work.
- Mean curve length (F34): Mean curve length was proposed with the purpose of reducing the complexity of Katz fractal dimension algorithm and provides results almost equivalent to it [33]. It is commonly used for identification of EEG activity, including amplitude and frequency changes and also its dimensionality [34]. Mean curve length, in its discrete form, is calculated using the following formula:
  11 $$ CL[n] = \sum\limits_{i = 1 + (n - 1)N}^{nN} {\left| {x(i) - x(i - 1)} \right|} $$
  considering *x* as the EEG data, *n* the epoch number and *N* the epoch length in samples.
- Hurst exponent (F35): Hurst exponent, introduced by Harold Edwin Hurst [35], is a measure for long range statistical dependence of time series. Hurst exponent has a value in the range between 0 and 1 and is defined as:
  12 $$ H = \frac{{\log \left( {{\raise0.7ex\hbox{$R$} \!\mathord{\left/ {\vphantom {R S}}\right.\kern-0pt} \!\lower0.7ex\hbox{$S$}}} \right)}}{\log (T)} $$
  where *T* is the duration of signal sample and $$ {\raise0.7ex\hbox{$R$} \!\mathord{\left/ {\vphantom {R S}}\right.\kern-0pt} \!\lower0.7ex\hbox{$S$}} $$ is the value of rescaled range.
- Itakura Spectral Distance (F36): The Itakura Spectral Distance (ISD) is broadly used in speech processing applications to measure the distance (similarity) between two auto regressive coefficients (AR) processes [36, 37]. ISD was also used in automatic sleep classification to find the relation between EEG and EOG signals during different epochs of sleep stages over the night [38]. In this paper, the ISD of sleep stages of EEG was measured. In order to calculate the distances, the AR coefficients were extracted from 50% of the wake epochs of each subject. Then, by getting the mean of the AR coefficients a representative model of the wake epoch was generated and the ISD between this model and the W (remaining 50%), S1, S2, SWS and REM epochs was calculated.
- Spectral power (F43): Power spectrum density (PSD) represents the distribution of signal’s power as a function of frequency. The spectral power of a signal in a frequency band is obtained by integrating PSD over the signal’s frequency range.

The physiological differences from subject to subject and equipment related variations have considerable impact on the features extracted from the PSG recordings. Moreover, since there are usually a wide variety of feature types extracted for characterizing sleep stages, the amplitude and unit of features will also vary. The features may also get the extreme values, i.e. extremely low or extremely high values. Data post-processing is an important step in this respect. The aim of feature post-processing is to enable classification algorithms to uniformly handle the features with different units and ranges as well as reducing the influence of extreme values. Feature post-processing can be a scaling operation (normalization/standardization) or a feature transformation operation. In this work, each feature (*x*_*ij*_) is independently scaled to have zero mean and unit variance $(x^{\prime}_{ij} )$ using the following equation:

13 $$ x^{\prime}_{ij} = \frac{{x_{ij} - {\bar{\mathbf{x}}}_{i} }}{{\sigma_{{{\mathbf{x}}_{{\mathbf{i}}} }} }} $$

where ${\bar{\mathbf{x}}}_{i}$ and $\sigma_{{{\mathbf{x}}_{i} }}$ are the mean and the standard deviation of each independent feature vector.

### Feature ranking methods

In this paper, to select a subset of features containing most of the original feature set information, we used seven different feature ranking methods: ReliefF, mini-mum redundancy-maximum relevance (MRMR-MID and MRMR-MIQ), Fisher score, Chi Square (Chi2), information gain (IG) and conditional mutual information maximization (CMIM). We have also implemented two different rank aggregation methods, Borda and robust rank aggregation (RRA), to evaluate their ability to produce better feature rankings compared to conventional feature ranking methods. A brief description of the used feature ranking methods is provided below:

#### ReliefF

In 1992, Kira and Rendell [39] proposed an instance based method, Relief, for estimating features quality. In this method, for a randomly selected sample, two nearest neighbors were considered: one from the same class (nearest *hit*) and other from a different class (nearest *miss*). The quality estimated for each feature is updated according to the randomly selected sample’s distance from the nearest hit and miss. The Relief method is restricted to two-class problems and is highly sensitive to noisy and incomplete data. An extension of Relief, called ReliefF [40], was proposed improving the original method by estimating the probabilities more reliably and extending the algorithm to multi-class problems. The ReliefF algorithm uses *k*-nearest hits and *k*-nearest misses for updating the quality estimation for each feature.

#### Minimum redundancy-maximum relevance

MRMR [41] is a feature selection method which selects a subset of features with maximum relevance for the target class and, at the same time, minimum redundancy between the selected features. In the MRMR method, the redundancy (R) and relevance (D) are expressed in terms of mutual information. In order to select the final feature set, an objective function *φ*(*D*, *R*) is maximized. The *φ*(*D*, *R*) can be defined either as the mutual information difference (MID), *D*-*R*, or the mutual information quotient (MIQ), *D/R*.

#### Fisher score

This method is one of the most efficient and widely used feature ranking methods. The key idea is to find a subset of the feature matrix with maximum distance between the data points from different classes and minimum distance between the data points of the same class in the feature space [42].

#### Chi square

Chi2 is another very common class sensitive feature selection method which ranks the features according to their Chi2 statistics without taking into account the interactions between features. Originally proposed exclusively for categorical data, this method was later extended to the continuous case [43]. For calculating the Chi2 statistics of each feature, the range of the numerical feature should be discretized into intervals.

#### Information gain

Ross Quinlan proposed an algorithm for generating decision trees from a set of training data [44]. In this algorithm, information gain (IG) is the measure for selecting the effective feature at each node. Generally, IG can be described as the change in the marginal entropy of a feature set taking into account the conditional entropy of that feature set with the given class set.

#### Conditional mutual information maximization

This method [45] is based on mutual information in such a way that all the selected features are informative and have two-by-two weak dependency. A feature is added to the selected feature subset if it contains information about the specific class and this information is not contained on any other previously selected feature.

#### Borda

The Borda algorithm is a feature aggregation method that ranks each feature based on its mean position in the different ranking methods considered, i.e.

14 $$ Borda(f_{i} ) = \sum\limits_{j = 1}^{N} {\pi_{j} (f_{i} )} $$

where *π*_*j*_(*f*_*i*_) is the rank of the feature *f*_*i*_ in the ranking method *π*_*j*_. The feature with the highest Borda rank is considered the best.

#### Robust rank aggregation

This method, proposed by Kolde et al. [46], is another rank aggregation method that compares the results from several feature ranking methods with a randomly ranked feature list. The RRA first looks how a specific feature is ranked by the various methods and lists the corresponding values in a so-called *rank order*, from best to worst. It is clear that, if a feature has high quality, the dominance of ranks in the rank order will be towards smaller numbers. The probability of the random list producing better ranking than the values seen in the actual rank order for that specific feature is determined. The features with the small probability are selected as the better ones [47].

### Classification

The process of labeling the data into relevant classes is called classification. The first step in the classification process is the identification of the features or characteristics that will enable the highest discrimination between the different groups of data. A classification model is developed in such a way that it provides the structure for how the classification processes’ actions will be realized. Ideally, this model should be chosen to optimize the performance of the classification system, although it may need to be revised as the classifier design progresses. A classifier is then implemented and “trained” to recognize the chosen features in the data, or to determine the best input-to-output mapping. Once the system has trained and learned, it is ready to classify specific inputs. Then, the system can be tested and evaluated with such metrics as speed of computation and accuracy of classification [48].

In this study, we selected two simple and widely used classifiers: *k*-nearest neighbor *(k*-NN) and multilayer feedforward neural network (MLFN) to discriminate five sleep stages W, S1, S2, SWS and REM. By selecting *k* = 1, nearest neighbor (NN) was utilized. The NN classifier is the simplest nonparametric classifier and assigns a pattern to a specific class based on its nearest neighbor’s class. In spite of its simplicity, in [49] it has been proved that, if the utilized database is fairly large, the error bound for nearest neighbor rule is quite tight, i.e. equal or less than twice the Bayes error. Also, neural networks are known to be very powerful computing models that can learn from training examples. Neural networks have been successfully used in a broad range of data mining applications including classification [50].

### Performance evaluation

In this paper three main criteria namely stability, accuracy and similarity are considered for evaluating and comparing the different feature selection techniques.

#### Stability

Stability of a feature selection method is defined as its sensitivity to variations in the training set. Since unstable feature selection may lead to inferior classification performance, a number of measures are proposed in the literature for investigating how different subsamples of a training set affect the feature importance assessment. In this study, in order to measure the stability of feature rankings produced by different methods, a similarity based approach proposed by Kalousis et al. [51] is used. In this method, similarity between two selected feature sets *s* and $s^{\prime}$, is calculated using the Tanimoto distance which measures the overlap between two sets of arbitrary cardinality:

15 $$ S_{s} (s,s^{\prime}) = 1 - \frac{{\left| s \right| + \left| {s^{\prime}} \right| - 2\left| {s \cap s^{\prime}} \right|}}{{\left| s \right| + \left| {s^{\prime}} \right| - \left| {s \cap s^{\prime}} \right|}} $$

The *S*_*s*_ takes values in the range of [0 1], with 0 meaning there is no overlap or similarity between two rankings and 1 meaning that the two rankings are identical. Then *N* subsets of the original training set are drawn using a random resampling technique such as cross validation or bootstrapping. Each specific ranking algorithm produces a feature preference list for each *N* subsets. The similarity between all possible pairs is calculated. The stability of that specific feature ranking algorithm is simply the average of the similarities over all possible pairs, i.e. $\frac{N(N - 1)}{2}$ pairs.

#### Similarity

The stability measure used for assessing the internal stability of a feature selection technique can also be used in a different context to measure the similarity of different feature selection techniques. The similarity measure provides information about the consistency and diversity of different feature selection algorithms. The similarity between two feature subsets *s* and $s^{\prime}$ can be calculated using Eq. (15) with a slight difference in the definition of *s* and $s^{\prime}$. Instead of two lists of features produced by a specific feature selection technique from different subsets of the training set, they are now two lists produced by two different feature selection techniques derived from the complete training set [51].

#### Accuracy

The performance of the sleep stage classification is evaluated using repeated random sub-sampling validation. To measure the classification accuracy, the overall accuracy value is calculated as follows [52]:

16 $$ {\text{Accuracy = }}\frac{{{\text{No}} . {\text{ of true detections}}}}{{{\text{Total no}} . {\text{ of epochs}}}} $$

### Experimental setup

Six subjects were selected from the Physionet database for evaluating and comparing the feature ranking and rank aggregation methods. For filtering EEG and EOG signals, Daubechies order 20 (db20) was used as the mother wavelet. The filtered data was segmented into 30-second epochs. From each epoch, a feature vector containing 49 features was extracted. After feature standardization, the feature vectors were fed into seven feature ranking methods. Then, in order to aggregate the results, the outputs of these seven feature ranking methods were used by Borda and RRA, producing two additional ranked lists of features.

For sleep stage classification, the parameters of the classifiers are set as follows. The Euclidean distance was chosen as the distance metric for the NN classifier. For the three-layer neural network classifier 12 hidden neurons and a sigmoid transfer function were selected in our simulations. The Levenberg–Marquardt training algorithm was adopted for minimizing the cost function because of its fast and stable convergence. In contrast with conventional approaches in the literature, which imports all the existing epochs to the classifier, we used a quantity of epochs selected out of each subject. In this method, selected epochs from each subject have two characteristics. Firstly, the number of epochs are the same for all the subjects. Second, the number of epochs for each stage is dependent on the number of occurrences of that stage for each subject. This method is suitable for large databases helping on the computational complexity reduction of the classifier training stage.

## Results

**Fig. 2:**
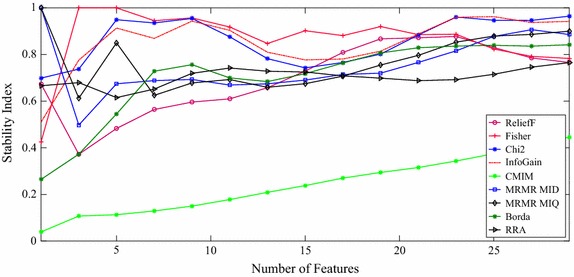
Stability measure of each feature selection method

The stability of each method was evaluated as a function of the number of selected features (*d*) where *d* = 1, 3, 5…29. In our simulations, 50 subsets were generated out of the original training set by bootstrapping. Figure 2 shows the stability of each method. In order to give an idea about the variations of stability in regard to the number of features, Table 4 provides significant information. In this table the mean value of stability is calculated for fifth, thirteenth and twenty-ninth features. Also, Table 5 illustrates the similarity between different feature selection methods. The similarity index has been calculated for the first 29 features selected by each method.

**Table 4:** Mean stability for selected features

	ReliefF	Fisher	Chi2	IG	CMIM	MRMR-MID	MRMR-MIQ	Borda	RRA
Mean stability up to 5th feature	0.50	0.80	0.79	0.73	0.20	0.72	*0.82*	0.39	0.65
Mean stability up to 13th feature	0.66	*0.99*	0.95	0.92	0.21	0.79	0.82	0.68	0.78
Mean stability up to 29th feature	0.69	0.86	0.86	*0.94*	0.24	0.75	0.77	0.70	0.70

Italic values indicate the maximum of each row

**Table 5:** Similarity of the feature selection techniques

	ReliefF	Fisher	Chi2	IG	CMIM	MRMR-MID	MRMR-MIQ	Borda	RRA
ReliefF	1	0.26	0.18	0.18	0.35	0.40	0.40	0.31	0.31
Fisher		1	0.58	0.52	*0.11*	0.58	0.65	0.72	0.65
Chi2			1	*0.90*	0.15	0.35	0.35	0.52	0.52
IG				1	0.18	0.35	0.35	0.46	0.46
CMIM					1	0.22	0.22	0.22	0.22
MRMR-MID						1	*0.90*	0.72	0.65
MRMR-MIQ							1	0.72	0.65
Borda								1	0.72
RRA									1

Italic values indicate the maximum and minimum similarity

**Fig. 3:**
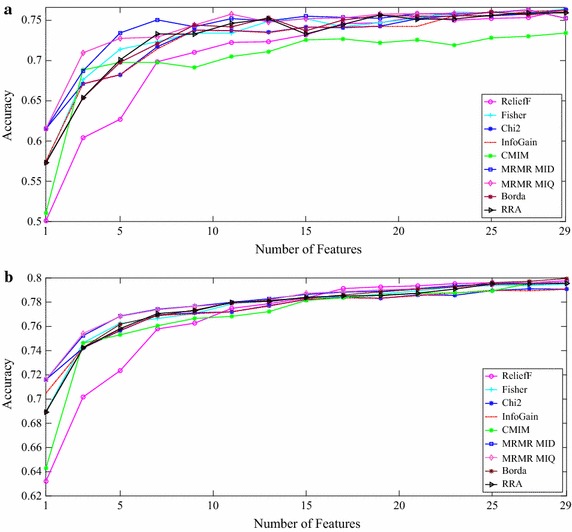
Classification accuracy for different feature selection methods. **a** *k*-NN classifier, **b** MLFN classifier

In order to estimate the generalization ability of the classifier, repeated random sub-sampling validation with 200 runs was applied. Figure 3 depicts the classification accuracy of *k*-NN and MLFN classifiers for different feature selection methods.

As Fig. 3 shows, starting with one feature, each additional feature typically leads to an increment in the classification accuracy. However, at some point, the increment on the classification accuracy for each additional feature is not significant leading to an elbow in the graph. Inspired by the “elbow” point in the cost-benefit curves, in this work we used the Kneedle algorithm proposed in [53] for determining the optimal feature number which provides a satisfactory trade-off between the selected number of features and the classification accuracy. Table 6 illustrates the top 10 features selected by each method.

**Table 6:** Top 10 features selected by each method and the optimum number selected by Kneedle algorithm (corresponding accuracy)

	ReliefF	Fisher	Chi2	IG	CMIM	MRMR-MID	MRMR-MIQ	Borda	RRA
Top 10 features	F28	*F36*	F35	F9	F15	F35	F35	*F36*	*F36*
	*F36*	F35	F9	F35	*F36*	F39	F42	F35	F35
	F7	F31	F11	F11	F9	*F36*	F15	F9	F9
	F49	F9	F31	F31	F8	F22	*F36*	F31	F31
	F41	F29	*F36*	*F36*	F1	F15	F22	F22	F27
	F27	F11	F27	F4	F34	F31	F23	F27	F22
	F20	F25	F26	F27	F35	F29	F31	F29	F17
	F23	F27	F4	F26	F28	F23	F38	F11	F29
	F6	F12	F25	F25	F6	F9	F29	F15	F11
	F22	F22	F14	F29	F48	F38	F9	F20	F20
MLFN	7 (0.75)	5 (0.76)	7 (0.76)	7 (0.76)	*3* (0.74)	5 (0.76)	5 (0.76)	5 (0.76)	7 (0.77)
*k*-NN	7 (0.69)	5 (0.71)	9 (0.73)	9 (0.73)	*3* (0.68)	7 (0.75)	11 (0.75)	9 (0.74)	7 (0.73)

Italic value indicates ISD feature

## Discussion

According to Fig. 2, Fisher method seems to have the highest stability and the CMIM method comes out to be the least stable one. Also, the stability of Chi2 and IG methods seems very convergent.

There exists a huge reduction in stability for MRMR_MID, MRMR_MIQ and ReliefF for three-feature subset, although after that stability increases slightly by each additional feature. Both MRMR methods are always 100% stable in selecting the first feature which is Hurst Exponent. It means that the Hurst Exponent has the highest discrimination ability from MRMR methods point of view. Also, the Fisher method has 100% stability for three-feature and five-feature subsets (ID, Hurst exponent, Petrosian fractal dimension as three-feature group and ID, Hurst exponent, Petrosian fractal dimension, zero-crossing rate and approximate entropy as five-feature group).

According to Table 4, MRMR-MIQ has the highest mean stability up to five features. Meanwhile, Fisher and Chi2 methods have almost the same stability value. Considering thirteen features, Fisher method is almost totally stable (99.92%). Finally, considering twenty-nine features, IG outperforms other methods from mean stability point of view.

According to Table 5, Chi2 and IG pair and MRMR-MID and MRMR-MIQ pair generate highly similar results. The similarity of MRMR methods can be explained by their similar theoretical background. On the contrary, CMIM and Fisher methods give the most dissimilar results. The average similarity of Borda and RRA methods is approximately 0.5 with the other methods. Regarding the aggregation characteristics it was predictable.

Table 6 illustrates the top 10 features selected by each method. ISD (F36) always appears in the top 10 for all the methods. In spite of the fact that different feature ranking methods have their own specific criteria for ranking the features, observing ISD in the top 10 list, means that ISD is a preferable feature for all the feature selection methods. In addition to ISD, there are some other features that can be considered most preferable according to Table 6. EEG ZCR (F9) is a simple, yet effective feature that is listed in top 10 by all of the methods except ReliefF. Following ZCR, Petrosian fractal dimension (F31), Hurst exponent (F35), WP feature (F22), approximate entropy (F29), spectral entropy (F27), and Hjorth mobility parameter (F11) are selected by at least five ranking methods to be included in top 10 list. On the other hand, features that are not in this list or are just selected by one method can be categorized as the least preferred features. EMG energy and energy ratio features (F47 to F49) and some of WP features are examples of least preferred features. The optimum number of features for each method, which is selected by the Kneedle algorithm, is shown in Table 6. For MLFN and *k*-NN classifiers, a slight difference exists in the optimum number. Considering the maximum accuracy that the methods reach in their optimum points, the MRMR-MID method using *k*-NN classifier outperforms all the others with seven selected features. Also, both MRMR methods using MLFN classifier outperform all the other methods with five features.

The CMIM method reaches its best accuracy with the first 3 features on both the classifiers. Considering Fig. 3, its accuracy is equal or less than the MRMR-MID method’s accuracy at that point. Unanticipatedly, none of the aggregation methods outperformed the rest of the feature ranking methods. One possible reason for this is that the aggregation methods, especially Borda, are affected by the performance of all the methods from best to worst.

## Conclusions and future works

In this paper we compared the performance of seven feature ranking methods for sleep stage classification. Feature selection based on filtering techniques has several advantages such as being fast, easily scalable to high-dimensional datasets, decrease computational complexity and work independently of the classifiers. Also, rank aggregation methods are supposed to be robust when used with a broad variety of classifiers and produce comparable classification accuracy to the individual feature selection methods. In this work, two rank aggregation methods were also applied to evaluate the performance on sleep stage classification. The Physionet Sleep-EDF Expanded Database was used to assess the impact of these methods on the classification accuracy of *k*-NN and MLFN. In addition, the stability and similarity of different feature selection methods were also evaluated. The results indicate that the MRMR-MID method slightly outperforms the other feature selection methods from the accuracy point of view. Considering that the CMIM produces the most unstable rankings, generally Fisher method produces the most stable results. When a small group of features (5–13) was required, the RRA aggregation method slightly outperformed the Borda. In our simulations, the performance of the aggregation methods was in the average level, although they are known to generate more stable results and better accuracy. It should be considered that the results presented in this paper are obtained through using Physionet Sleep-EDF Expanded Database which is already used in several previous sleep studies [19, 54–56] and can be supposed as verified enough to be used in such a comparative study. Nevertheless, generalizing these results to all future sleep studies requires further study and analysis by using other sleep databases as well. Also, in this paper for evaluating the generalization ability of classifiers we used repeated random subsampling validation. In [57], it is mentioned that due to the data subdivision dependency resulted from validation methods that are based on random subsampling, patient cross validation was preferred. Therefore, future steps will involve verifying the results with different databases, applying and comparing more rank aggregation methods and also using patient cross validation and comparing the results with common validation methods.
